# Supplementary material for: Two melanic pigment patterns are associated with a sex chromosome-linked oncogene in the mountain swordtail Xiphophorus nezahualcoyotl
Source: bioRxiv. 2026 Jun 9:2026.06.08.730778. Preprint. [Version 1] doi: 10.64898/2026.06.08.730778 (PMC13277934; doi:10.64898/2026.06.08.730778)
Supplement: Supplementary file 1 [file media-1.pdf]

Supplemental Information

Supplementary Text

Choice of reference genome and read mismapping artifacts

Long-read sequencing enables assembly and analysis of complex genomic regions [1]; however, its current high cost restricts implementation at population-level scales, particularly in non-model species. Here, we employed a mixed-sequencing approach, where long-read sequencing is performed on a subset of individuals to generate high-quality genome assemblies, and short-read sequencing is performed on the remaining individuals for reference-based analyses requiring larger samples (e.g. GWAS). However, errors can arise when mapping short-read sequences to polymorphic and complex regions [2]. One well-documented artifact is reference bias, which occurs when reads matching the reference genome map more readily to reference alleles than reads containing alternate alleles [3]. In population-scale studies, this can cause over-representation of reference alleles and under-representation of alternate alleles as variants present in the population but absent in the reference genome are missed [3–5].

A perhaps less-appreciated phenomenon that has gained some recent attention is artifacts derived from read mismapping due to true differences in sequence composition between the reference sequence and GWAS panel [1,6,7]. In principle, if portions of the genome are present in an individual selected for whole-genome resequencing, but the homologous region is absent from the reference genome, the sequence may nonetheless map to a non-homologous region if it is similar. In cases where the non-homologous region is single- or low-copy in the reference genome, reads may not be filtered out using standard approaches that rely on mapping quality scores (mapQ) or standard mappability filters. If downstream analyses rely on variant calls derived from these regions, spurious associations may occur. Read mismapping has been discussed in several contexts, including in eQTL [6] and microbiome analyses [1]. However, its contribution to GWAS with respect to trait variation in complex regions is less well understood.

To investigate the implications of reference choice when mapping short-reads to complex genomic regions, we conducted GWAS for the two spotting patterns and sex using each long-read assembly pseudohaplotype (n=10) that we generated in this study. In the main text, we report results of GWAS using the primary assembly from the near-T2T female (Fig 3) for which chromosome 21 is a T2T X chromosome that contains *xmrk*. In the supplement, we report and qualitatively compare outcomes of GWAS using references derived from the psuedohaplotypes generated from individuals with diverse phenotypes. In these instances, chromosome 21 may be an X chromosome (Fig. S7) or a Y chromosome (Fig. S8) and may or may not contain *xmrk*.

Results of GWAS for spotting patterns to alternate references vary substantially. We identified a trend that, when *xmrk* is absent in a reference genome, a significant association was often identified with its paralog *egfrb* (Fig. S7,S8), which occurs >2 Mb away on chromosome 21 but has >96% coding sequence identity. We discuss several examples to illustrate this trend.

When a reference containing *xmrk* is used (Ss-M01-h1; Fig. S7a), there was a single significant association near *xmrk* (similar to the reference in the main text; Fig 3). However, when the reference assembly lacks *xmrk*, as was true in many cases, we observed one of several possible outcomes. In some instances, the reference contains two GWAS peaks (e.g., Ss-M02-h2; Fig. S7b), one near positions flanking where *xmrk* typically. In other cases, there only a peak at *egfrb* and no evidence of other signal (e.g., Ma-M01-h2). Compared to GWAS with spotted side, marmoratus displayed a weaker signal near *egfrb*, often with only a single associated SNP. Broad trends on the X chromosomes were similar when we conducted GWAS to assemblies containing the Y chromosomes from diverse phenotypes (Fig. S8) although the peak SNPs near *xmrk* were sometimes less significantly associated.

While we do not think the inconsistency brought from using reference genomes without the gene of interest weakens the conclusion of this work that *xmrk* is associated with spotted phenotypes, these artifacts are certainly notable and a cause for concern. The case of the near-T2T female (Ss-F01), which is hemizygous for *xmrk,* is particularly illustrative. Only GWAS with the primary assembly (or hap1) containing *xmrk* identified a significant association with *xmrk*. If hap2 had been assigned as the primary assembly, and if we had not run GWAS using multiple haplotypes, it is possible this association with *xmrk* would not have been discovered. We note there has been over three decades of research on *xmrk* and a long controversy surrounding its role as Mdl, which is an unusually substantial body of work for a lineage-specific gene in a non-model species. This highlights potential challenges in identifying the drivers of associations in cases where the underlying genetic architecture is a presence-absence variant in a structurally challenging region. Recent work has proposed that the use of pangenome graphs may alleviate this issue by reducing mismapping rates [7]. However, pangenome approaches may be more limited in complex regions of the genome, such as the sex chromosomes. Thus, we urge researchers to consider how the selection of the reference genome can have large impacts on GWAS results, and suggest that further work is needed to improve tools and approaches for challenging genomic regions.

Table S1. Long-read sequencing statistics for all samples. We generated between 30x and 80x coverage of PacBio HiFi or Oxford Nanopore Technologies long-read data for individuals of varying phenotypes.

Table S1. Long-read sequencing statistics for all samples. We generated between 30x and 80x coverage of PacBio HiFi or Oxford Nanopore Technologies long-read data for individuals of varying phenotypes.

Species	Alias	Source	Sex	Phenotype	Technology	Data (Gb)	Coverage	Read N50	Median Read QV
X. nezahualcoyotl	Ss-F01	PTES; Wild caught	F	Spotted side (Ss)	ONT UL	57.768	80.79x	57,716	21.762
X. nezahualcoyotl	Ma-M01	PTES; Wild caught	M	Marmoratus (Ma)	PacBio HiFi	32.021	44.78x	22,239	31.169
X. nezahualcoyotl	WT-M01	TANC; Lab born	M	Unspotted (WT)	PacBio HiFi	33.171	46.39x	16,283	32.013
X. nezahualcoyotl	Ss-M01	TANC; Lab born	M	Spotted side (Ss)	PacBio HiFi	22.043	30.83x	22,482	30.655
X. nezahualcoyotl	Ss-M02	TANC; Lab born	M	Spotted side (Ss)	PacBio HiFi	34.365	48.06x	21,077	32.306
					ONT UL	32.510	45.47x	16,022	24.245

Table S2. Long-read assembly statistics. Contiguity (contig/scaffold N50, contig/scaffold number, gaps, % basepairs contained in longest 24 scaffolds, and number of T2T contigs), completeness (BUSCO), and correctness (Merqury QV) metrics were calculated for the primary near-T2T assembly following manual curation, as well as for both pseudohaplotypes assembled by hifiasm. Note that the mitochondrion was included in the curated near-T2T assembly. BUSCO was run on the actinopterygii odb12 database. Abbreviations: C – complete; S – complete, single copy; D – compete, duplicated; F – fragmented; M – missing; n – number.

**Assembly**	**Alias**	**Assembly size (Mb)**	**Contig N50 (Mb)**	**# contigs**	**Scaffold N50**	**# scaffolds**	**Gaps**	**% in longest 24 scaffolds**	**T2T contigs**	**BUSCO (genome mode)**	**Merqury QV**
Primary, curated	Ss-F01 near-T2T	715.212	32.000	25	32.000	25	0	99.998	22	C:99.4%[S:99.1%,D:0.3%],F:0.1%,M:0.5%,n:7207	74.3946
Ss-F01 Haplotype 1	Ss-F01-h1	715.062	31.967	32	31.967	30	2	99.82	21	C:99.4%[S:99.0%,D:0.4%],F:0.1%,M:0.5%,n:7207	74.2353
Ss-F01 Haplotype 2	Ss-F01-h2	713.659	30.574	32	31.967	28	4	99.96	20	C:99.4%[S:98.9%,D:0.5%],F:0.2%,M:0.5%,n:7207	78.1398
Ma-M01 Haplotype 1	Ma-M01-h1	713.458	28.966	81	31.286	66	15	99.16	6	C:99.3%[S:98.9%,D:0.4%],F:0.1%,M:0.6%,n:7207	68.7963
Ma-M01 Haplotype 2	Ma-M01-h2	712.232	29.814	81	31.821	53	28	99.25	3	C:99.3%[S:98.8%,D:0.5%],F:0.2%,M:0.5%,n:7207	73.278
WT-M01 Haplotype 1	WT-M01-h1	714.633	28.830	87	31.599	74	13	99.41	8	C:99.3%[S:98.9%,D:0.4%],F:0.1%,M:0.6%,n:7207	70.4548
WT-M01 Haplotype 2	WT-M01-h2	714.861	28.089	93	31.842	66	27	99.40	7	C:99.4%[S:98.9%,D:0.5%],F:0.1%,M:0.5%,n:7207	70.0888
Ss-M01 Haplotype 1	Ss-M01-h1	713.331	24.745	89	30.008	50	39	99.57	3	C:99.4%[S:99.0%,D:0.3%],F:0.1%,M:0.5%,n:7207	69.4535
Ss-M01 Haplotype 2	Ss-M01-h2	714.046	26.824	74	30.068	49	25	99.40	4	C:99.2%[S:98.8%,D:0.4%],F:0.2%,M:0.6%,n:7207	71.817
Ss-M02 Haplotype 1	Ss-M02-h1	715.514	29.801	54	32.006	48	6	99.56	12	C:99.4%[S:98.9%,D:0.4%],F:0.2%,M:0.5%,n:7207	73.8304
Ss-M02 Haplotype 2	Ss-M02-h2	707.984	29.941	57	29.960	47	10	99.42	8	C:98.7%[S:98.3%,D:0.4%],F:0.2%,M:1.2%,n:7207	71.9847

Table S3. Assessment of LiftOn annotation quality using BUSCO. BUSCO scores for predicted proteins based on NCBI annotation for *Xiphophorus maculatus* and for predicted proteins in *X. nezahualcoyotl* using LiftOn annotations. Most BUSCO genes annotated in *X. maculatus* have been successfully lifted over to *X. nezahualcoyotl.* BUSCO abbreviations: C – complete; S – complete, single copy; D – compete, duplicated; F – fragmented; M – missing; n – number.

**Alias**	**Annotation method**	**BUSCO (protein mode)**
GCF_002775205.1_X_maculatus-5.0-male	Gnomon	C:99.0%[S:98.5%,D:0.5%],F:0.4%,M:0.6%,n:7207
Ss-F01-h1	LiftON	C:98.6%[S:98.1%,D:0.5%],F:0.6%,M:0.8%,n:7207
Ss-F01-h2	LiftON	C:98.7%[S:98.2%,D:0.5%],F:0.5%,M:0.8%,n:7207
Ma-M01-h1	LiftON	C:98.7%[S:98.2%,D:0.5%],F:0.5%,M:0.8%,n:7207
Ma-M01-h2	LiftON	C:98.7%[S:98.1%,D:0.6%],F:0.5%,M:0.8%,n:7207
WT-M01-h1	LiftON	C:98.6%[S:98.2%,D:0.4%],F:0.5%,M:0.9%,n:7207
WT-M01-h2	LiftON	C:98.7%[S:98.2%,D:0.5%],F:0.5%,M:0.8%,n:7207
Ss-M01-h1	LiftON	C:98.7%[S:98.3%,D:0.4%],F:0.5%,M:0.8%,n:7207
Ss-M01-h2	LiftON	C:98.6%[S:98.1%,D:0.5%],F:0.5%,M:0.9%,n:7207
Ss-M02-h1	LiftON	C:98.8%[S:98.3%,D:0.5%],F:0.4%,M:0.7%,n:7207
Ss-M02-h2	LiftON	C:98.1%[S:97.7%,D:0.4%],F:0.5%,M:1.4%,n:7207

Table S4. Annotation of each assembly with pan-TE libraries. Percent of genome annotated as repeat content (bases masked) for each long-read pseudo-haplotype using two libraries, Pantera (polymorphic elements) or Pantera plus LTR elements (polymorphic elements and fixed LTRs).

Assembly	% masked with Pantera library	% masked with Pantera + LTR library
Ss-F01-h1	19.73	22.89
Ss-F01-h2	19.79	22.94
Ma-M01-h1	19.76	22.91
Ma-M01-h2	19.59	22.75
WT-M01-h1	19.83	22.97
WT-M01-h2	19.71	22.86
Ss-M01-h1	19.68	22.83
Ss-M01-h2	19.82	23.00
Ss-M02-h1	19.86	23.03
Ss-M02-h2	19.7	22.83

Table S5. Locations with potential errors on chromosome 21 identified by Merqury. Kmer-containing intervals were merged by bedtools if within 1000 bp. Most errors occur within the first and last 100 kb of the chromosome. We detected no errors in the sex-linked region that was analyzed for structural variation the main text.

Chr-21 assembly	Region start	Region stop	Error type
Ss-F01-h1#21	12755824	12755855	Merqury
Ss-F01-h2#21	19745807	19745832	Merqury
Ma-M01-h1#21	1943844	1943877	Merqury
Ma-M01-h2#21	NA	NA	NA
Ss-M01-h1#21	NA	NA	NA
Ss-M01-h2#21	1595438	1595478	Merqury
Ss-M01-h2#21	27500078	27500508	Merqury
Ss-M02-h1#21	41	2875	Merqury
Ss-M02-h1#21	7581	8252	Merqury
Ss-M02-h1#21	9741	9769	Merqury
Ss-M02-h1#21	11112	11433	Merqury
Ss-M02-h1#21	47232	47262	Merqury
Ss-M02-h1#21	2534467	2534492	Merqury
Ss-M02-h1#21	6849462	6849498	Merqury
Ss-M02-h1#21	27482710	27482750	Merqury
Ss-M02-h2#21	0	1923	Merqury
Ss-M02-h2#21	35031	35061	Merqury
Ss-M02-h2#21	15485093	15485132	Merqury
Ss-M02-h2#21	26569876	26569910	Merqury
Ss-M02-h2#21	26576327	26576368	Merqury
WT-M01-h1#21	28199444	28199480	Merqury
WT-M01-h2#21	NA	NA	NA
Ss-F01-h1#21	12755824	12755855	Merqury
Ss-F01-h2#21	19745807	19745832	Merqury
Ma-M01-h1#21	1943844	1943877	Merqury
Ma-M01-h2#21	NA	NA	NA

Table S6. NCBI accessions for outgroup sequences used in phylogenetic analysis. Gambusia affinis, a relative with a high quality reference genome, was used as the outgroup for all phylogenetic analyses.

Species	Gene	Biotype	NCBI accession
Gambusia affinis	*egfrb*	mRNA	XM_044104981.1
Gambusia affinis	*egfrb*	protein	XP_043960916.1

Table S7. Genome assemblies used to construct *Xiphophorus* Pantera and LTR repeat databases. To broaden use of this repeat database and capture elements that may be fixed in *X. nezahualcoyotl*, we constructed repeat databases using available Xiphophorus genomes.

Species	Assembly	Sex	Site	Technology	Publication	Status
X. birchmanni	xbir-COAC-S238-19-IX-23-M01	M	Rio Coacuilco	PacBio HiFi	Dodge et al. 2024	finalized
X. continens	xcon-NROF-20-III-25-M01	M	Nacimiento de Rio Ojo Frio	PacBio HiFi	unpublished	draft
X. cortezi	xcor-PTHC-08-XII-21-M	M	Rio Huicihuyan	PacBio HiFi	Langdon et al. 2024	finalized
X. evelynae	xeve-JUCH-S178-IX-23-M01	M	Juntas Chicas	PacBio HiFi	Haghani et al. 2025	finalized
X. malinche	xmal-CHIC-XI-20-M	M	Chicayotla	PacBio HiFi	Dodge et al. 2024	finalized
X. montezumae	xmoz-ROJF-17-III-25-M02	M	Rio Ojo Frio	PacBio HiFi	unpublished	draft
X. multilineatus	xmul-TABQ-S253-5-XI-24-M01	M	Rio Tambaque	PacBio HiFi	unpublished	draft
X. nezahualcoyotl	xnez-TANC-19-I-22-M	M	Rio Tanchachin	PacBio HiFi	This work	finalized
X. nigrensis	xnig-C-NARC-18-III-25-M01	M	Nacimiento de Rio Choy	PacBio HiFi	unpublished	draft
X. pygmaeus	xpyg-PTHC-S063-7-VIII-23-M01	M	Rio Huicihuyan	PacBio HiFi	unpublished	draft
X. variatus	xvar-COAC-S280-21-XI-24-M01	M	Rio Coacuilco	PacBio HiFi + ONT	unpublished	draft

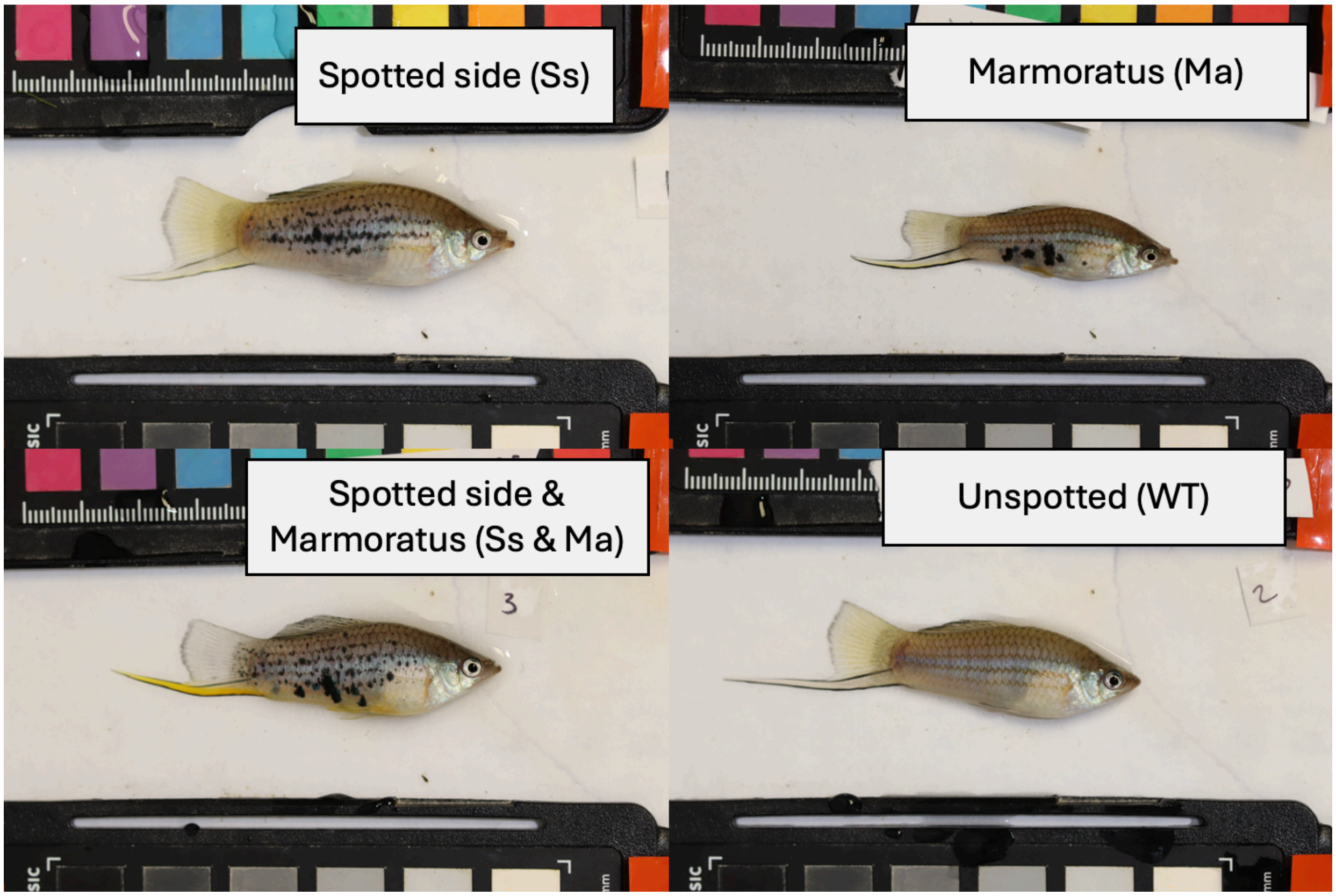

Figure S1. Examples of *X. nezahualcoyotl* males with different spotting patterns. Top left has spotted side (Ss), top right has marmoratus (Ma), bottom left is a male with both spotting patterns (Ss and Ma), and bottom right is unspotted (WT).

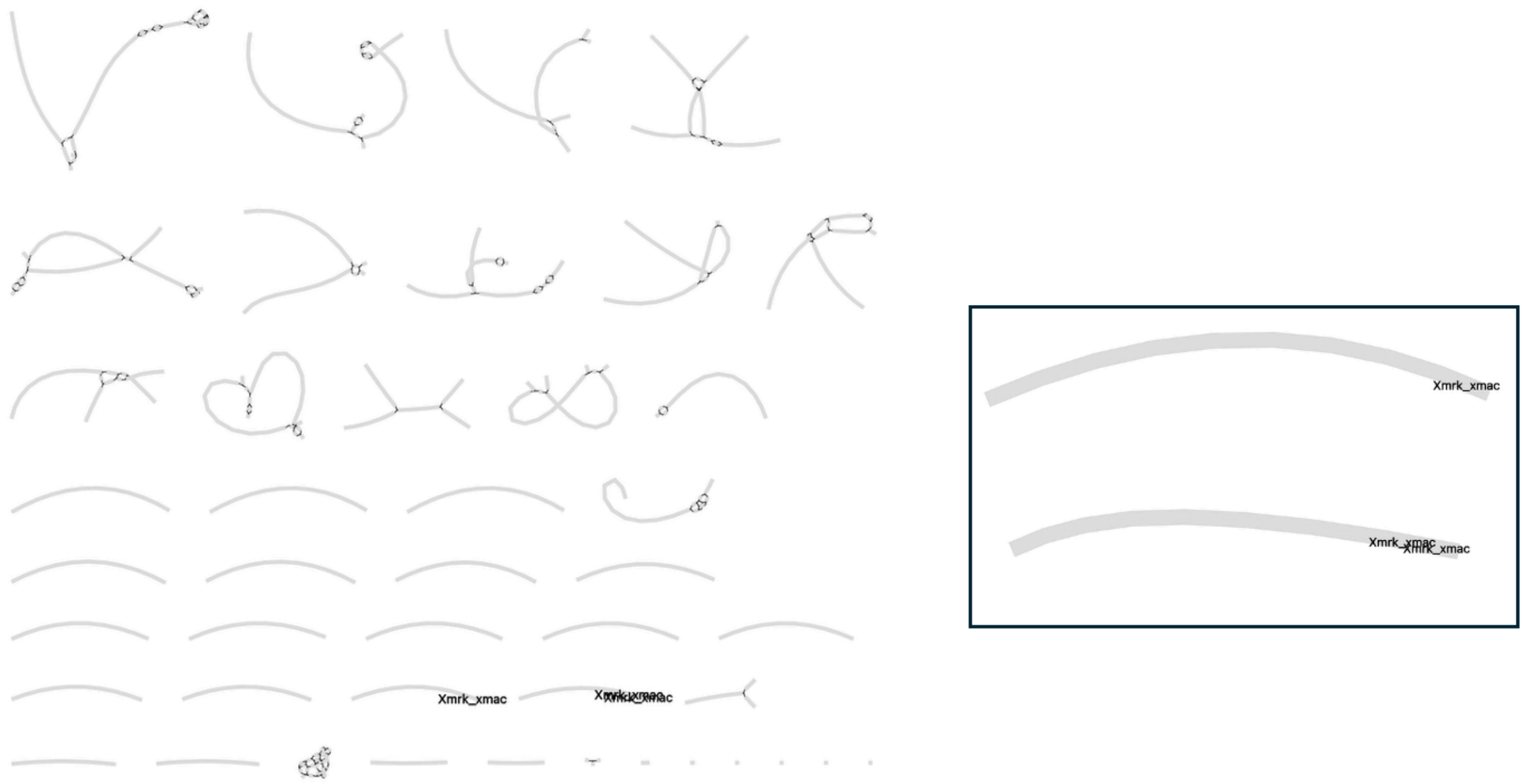

Figure S2. Phased diploid assembly graph using ONT ultralong data. Hifiasm unitig assembly graph for near-T2T assembly (left) and for chromosome 21 (right, black box). Blast results of *xmrk* from *X. maculatus* are shown, with one unitig containing a single hit (*egfrb*) and one unitig containing two blast hits (*egfrb* and a putative *xmrk*). Several chromosomes, including chromosome 21 (the chromosome containing the blast hits), are completely phased in the processed hifiasm unitig graph.

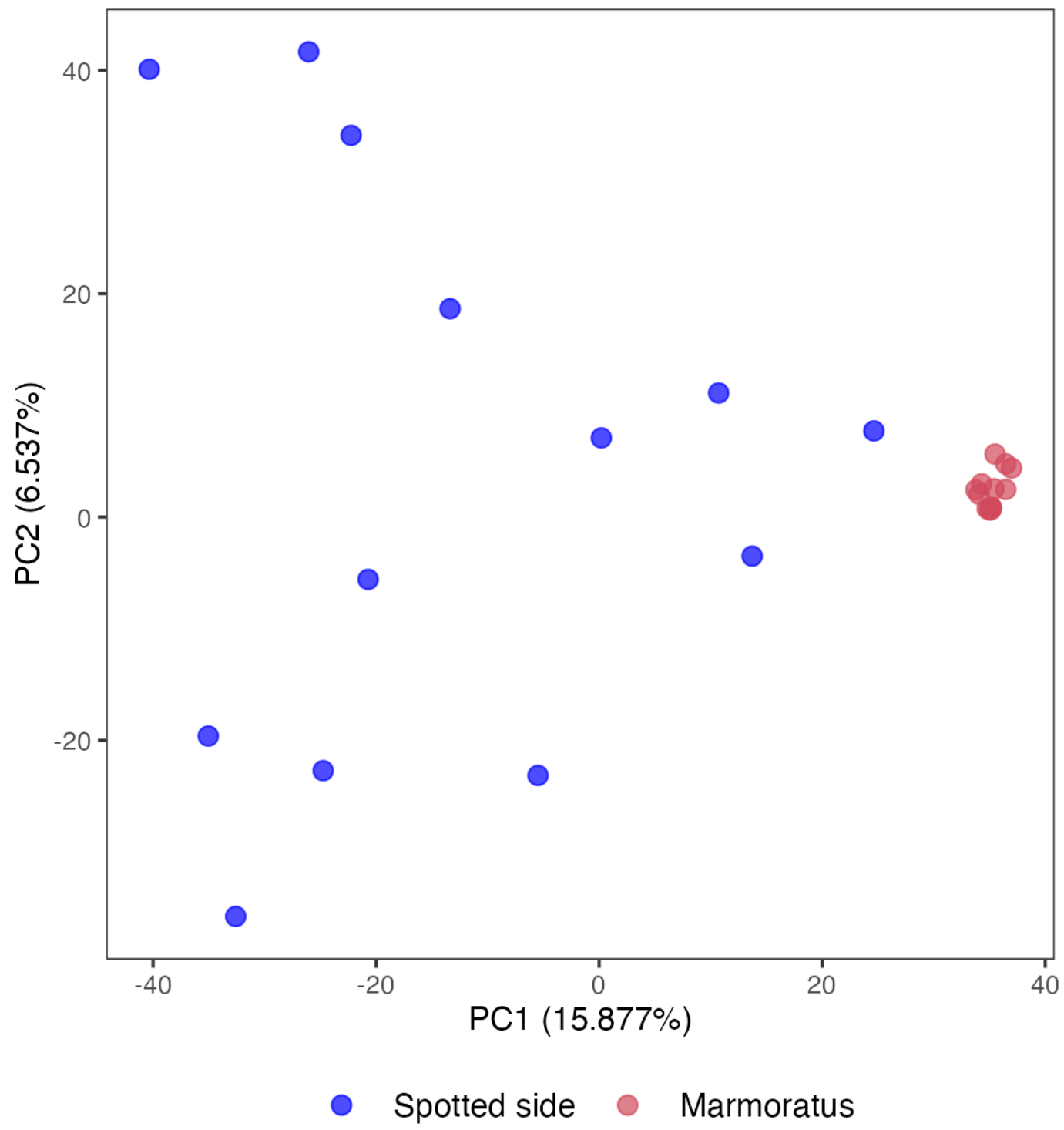

Figure S3. Downsampled Phenotype PCA. The pattern dataset was downsampled to include all individuals with marmoratus (n=13), and an equal number of individuals with spotted side (n=13). The separation between phenotypic clusters, and separation of the spotted side phenotype relative to marmoratus along PC2 persists after downsampling.

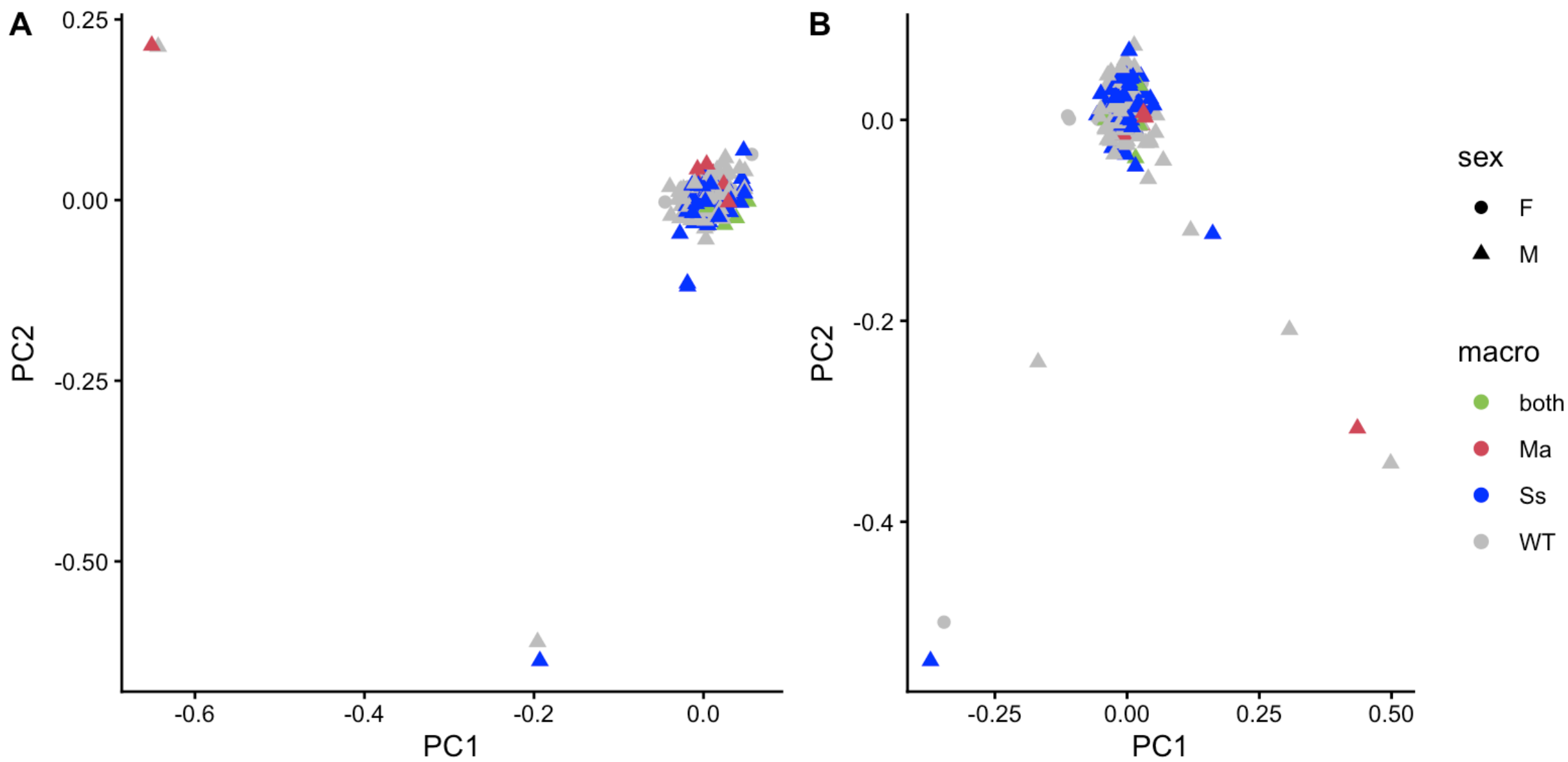

Figure S4. Genomic PCA from the population of *X. nezahualcoyotl* used for GWAS. We found no evidence that population structure was correlated with phenotype in this *X. nezahualcoyotl* population. 6 individuals, which represented individuals across all macromelanophore phenotype classes, appeared to be outliers. We repeated the PCA after excluding these individuals and still did not detect population structure correlated with phenotype. Note that there are substantially more males in the GWAS dataset than there are females. Ss – Spotted side; Ma – Marmoratus; WT – Unspotted.

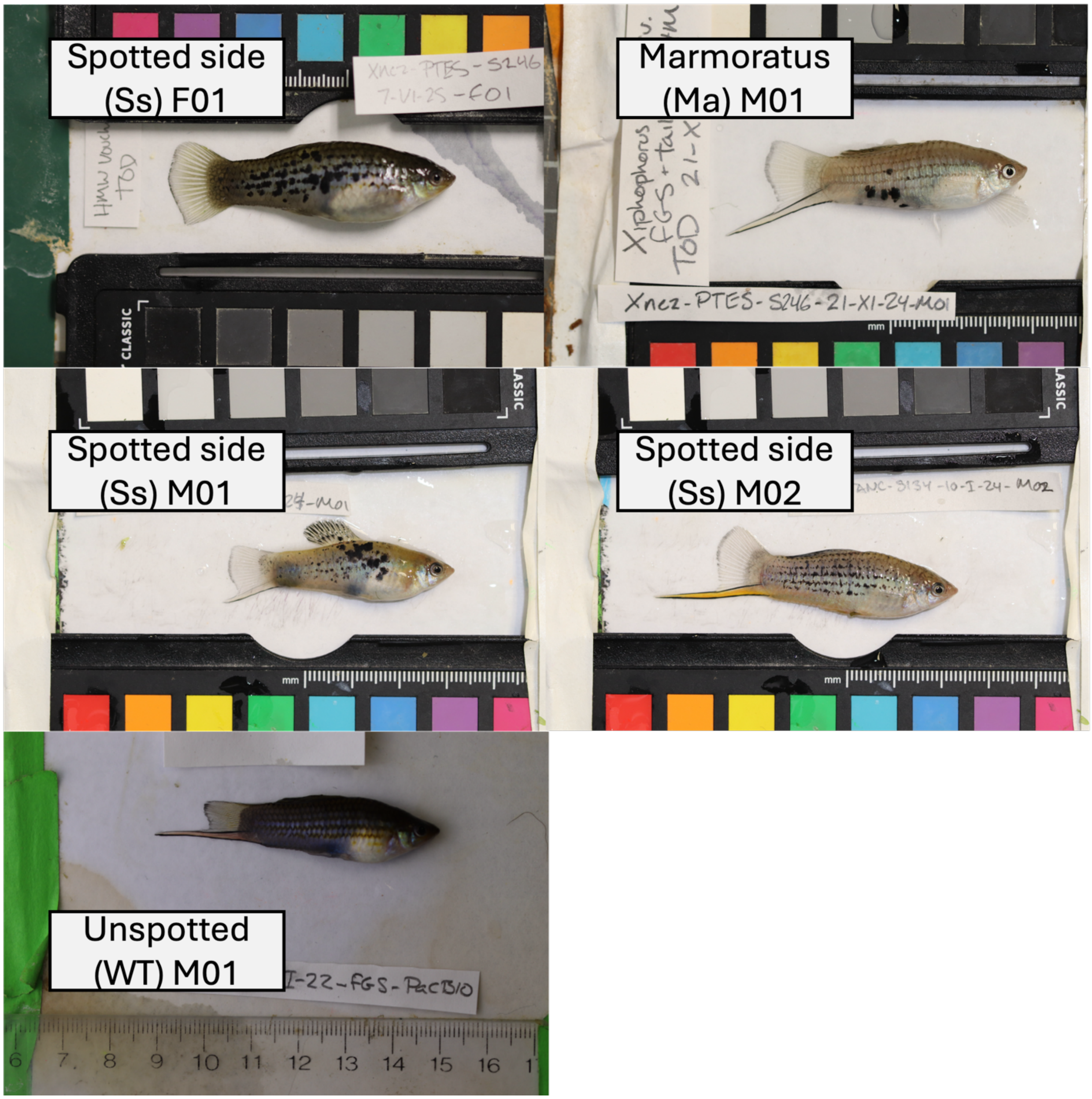

Figure S5. Photos of the five *X. nezahualcoyotl* individuals used to produce long-read assemblies. Top left is the near-T2T female with spotted side (Ss-F01), top right is the male with marmoratus (Ma-M01), middle are the spotted side males (Ss-M01 and Ss-M02), and bottom left is the unspotted male (WT-M01). Note that lighting conditions are not standardized between photographs, and WT-M01 was photographed before flash was implemented as part of the standard operating procedure.

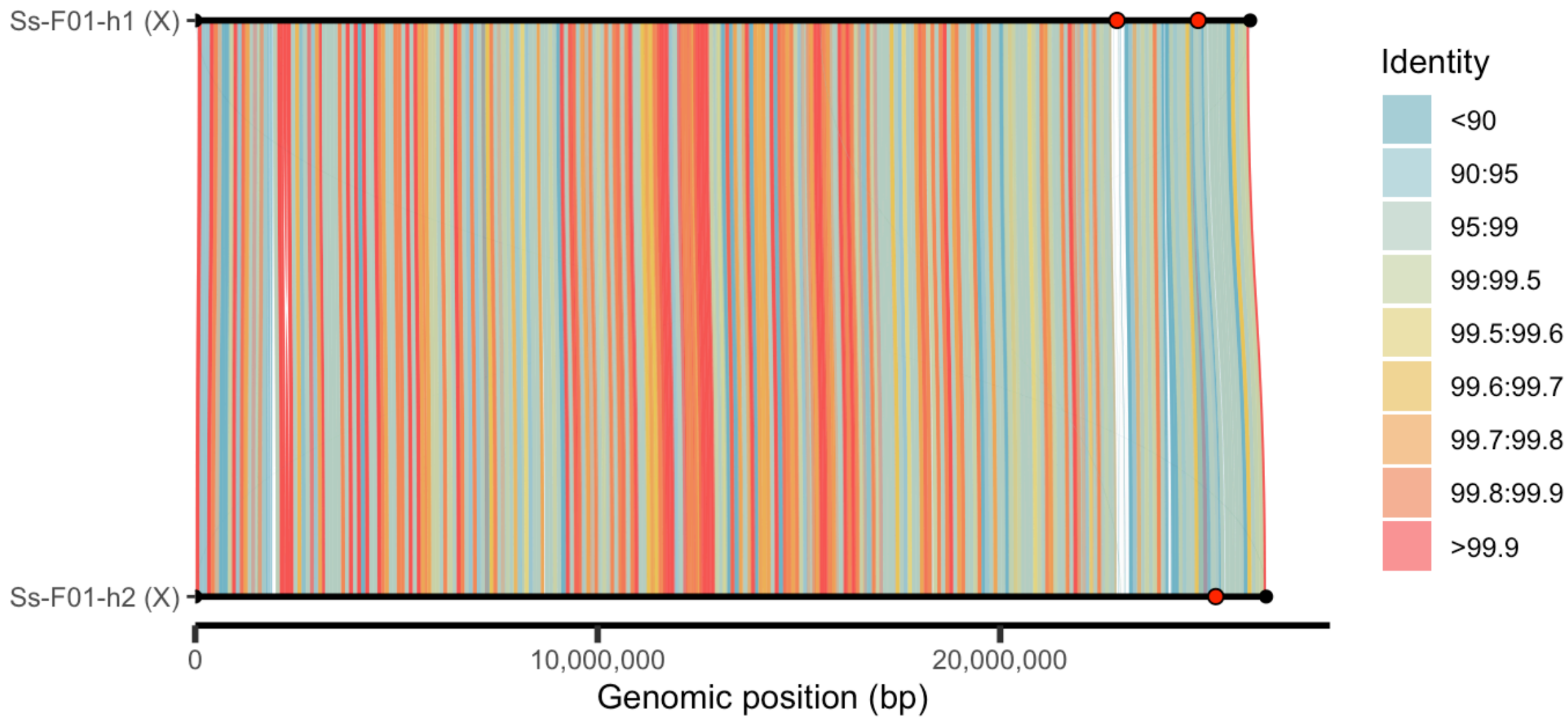

Figure S6. SVbyEye alignments of gapless *X. nezahualcoyotl* X chromosomes. Red dots represent *xmrk*/*egfrb* blast hits and black dots represent telomeres. The region containing *xmrk* does not align between haplotypes (white). Alignments are colored by percent identity in 100 kb bins. Note that these percent identity calculations treat gaps in the alignment as mismatched basepairs.

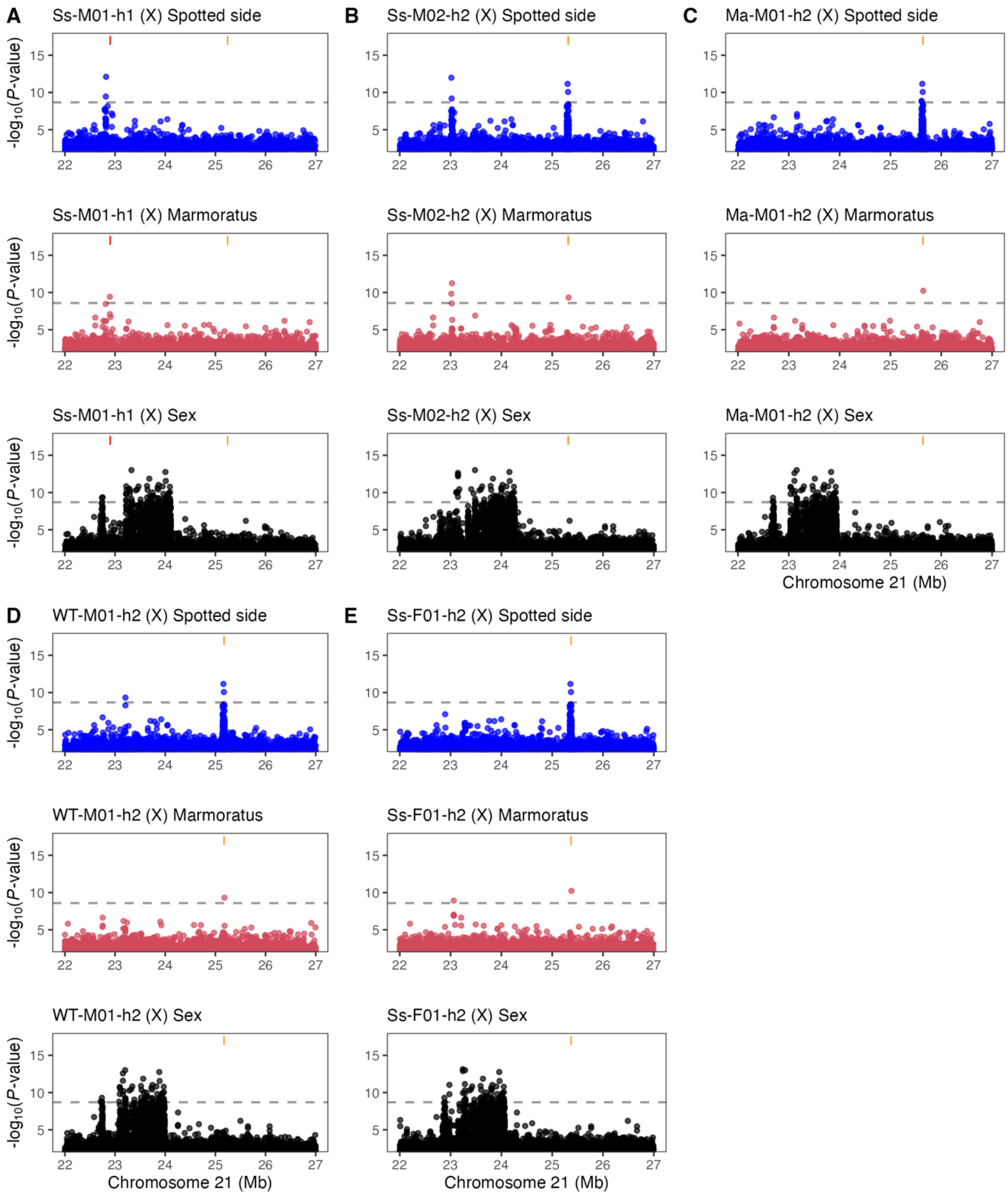

Figure S7. GWAS results from analyses mapping data to different reference genomes, focusing on the pseudohaplotype that contains the X chromosome. All plots span from position 22 to 27 Mb on chromosome 21, and the y-axis shows -$\log_{10}$(p-value). Blue Manhattan plots are GWAS results for spotted side, pink are for marmoratus, and black are for sex. The reference haplotype **(a)** Ss-M01-h1 contains both *xmrk* (red) and *egfrb* (orange), and the

remaining haplotypes **(b)** Ss-M02-h2, **(c)** Ma-M01-h2, **(d)** WT-M01-h2, and **(e)** Ss-F01-h2 only contain *egfrb* (orange).

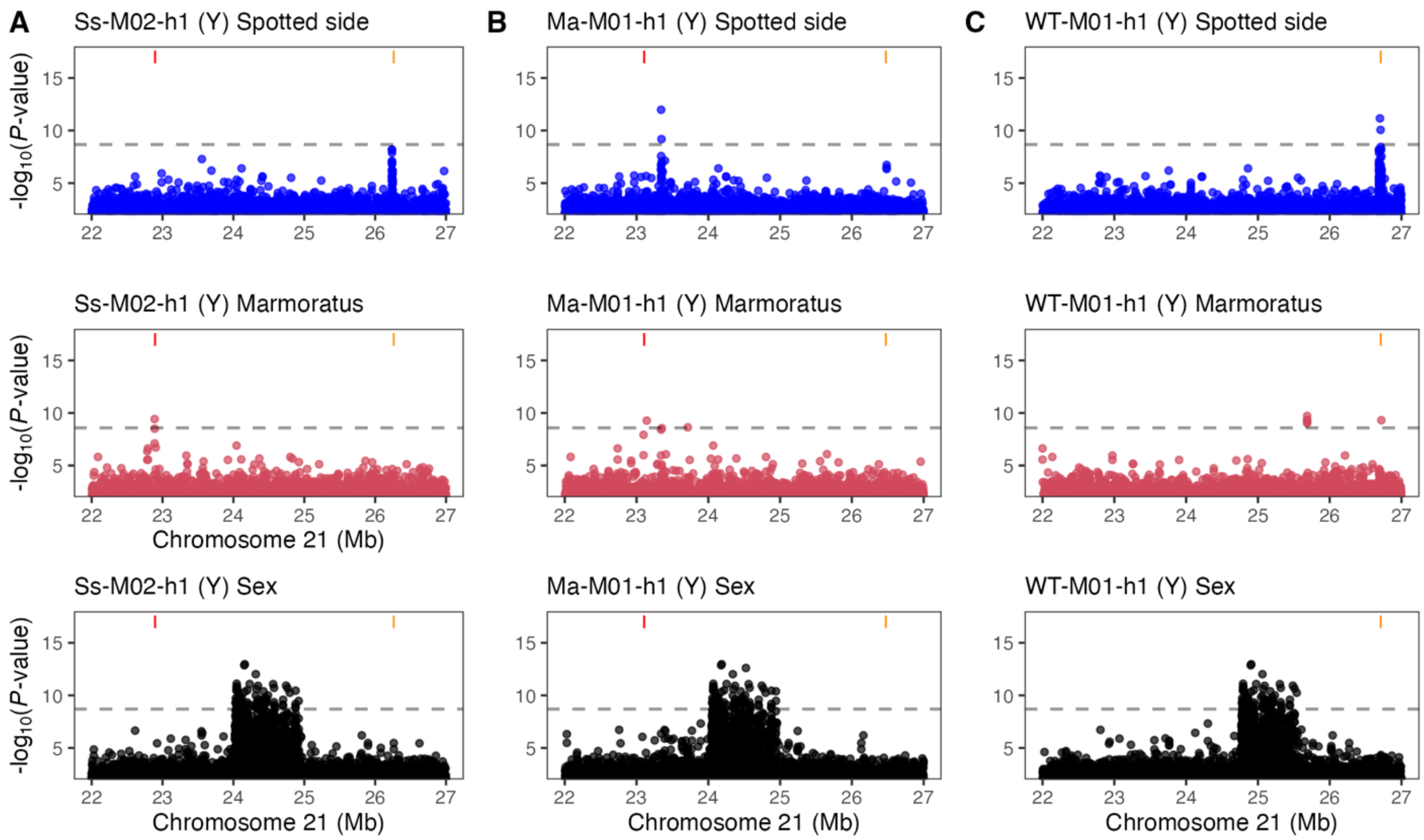

Figure S8. GWAS results from analyses mapping data to different reference genomes, using the pseudohaplotype that contains the Y chromosome. All plots span from position 22 to 27 Mb on chromosome 21, and the y-axis shows -$\log_{10}$(p-value). Blue Manhattan plots are GWAS results for spotted side, pink are for marmoratus, and black are for sex. The reference haplotypes Ss-M02-h1 **(a)** and Ma-M01-h1 **(b)** contain both *xmrk* (red) and *egfrb* (orange), and the reference haplotype WT-M01-h1 **(c)** only contains *egfrb* (orange).

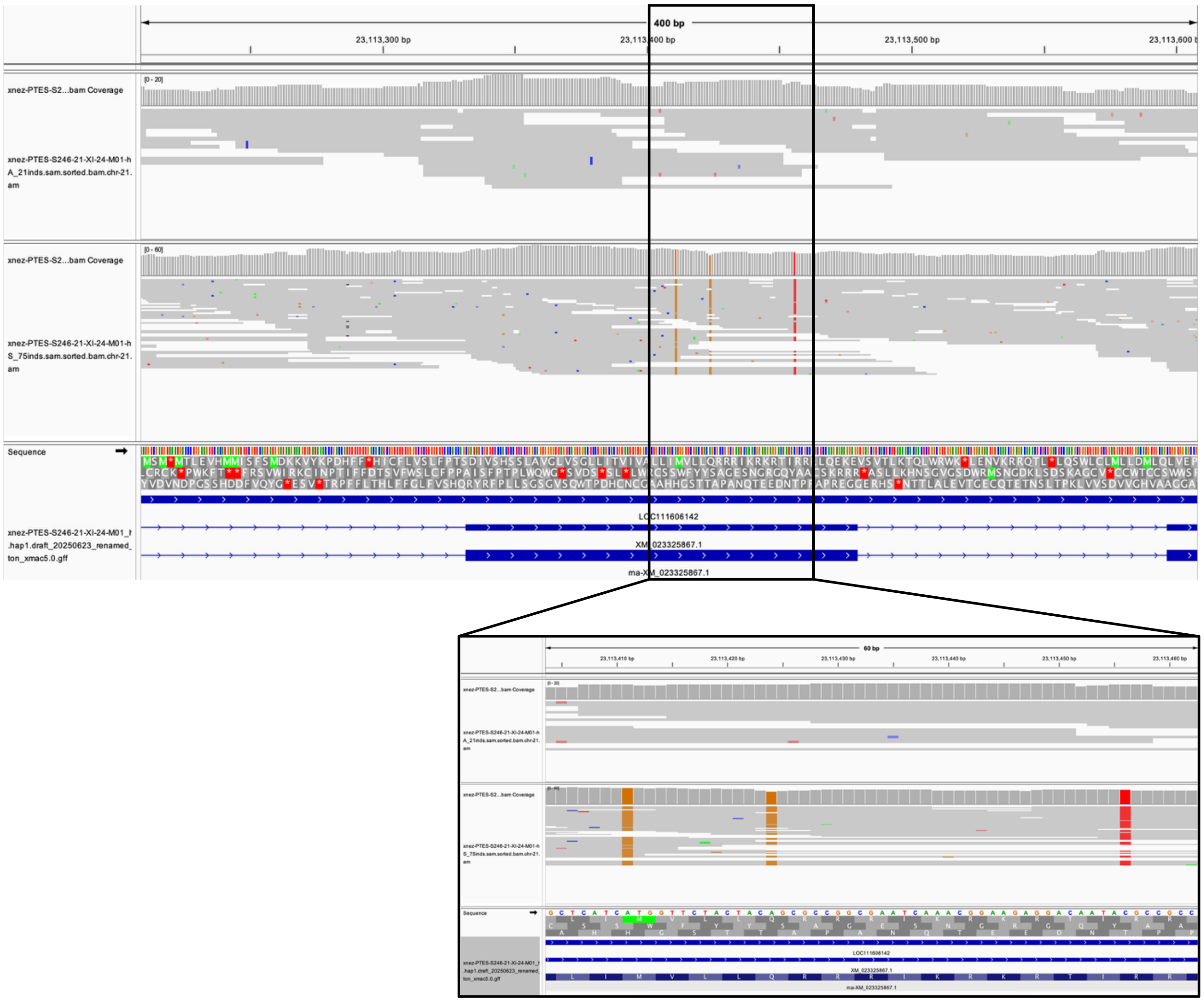

Figure S9. Derived SNPs in marmoratus *xmrk* appear fixed in analysis of pooled sequencing data from marmoratus individuals. Integrative Genomics Viewer showing *xmrk* sequences from pooled whole-genome short-read data mapped to the marmoratus Y chromosome. The three non-synonymous SNPs identified in the marmoratus and spotted side long-read assemblies appear to be fixed in each haplotype at the population-level.

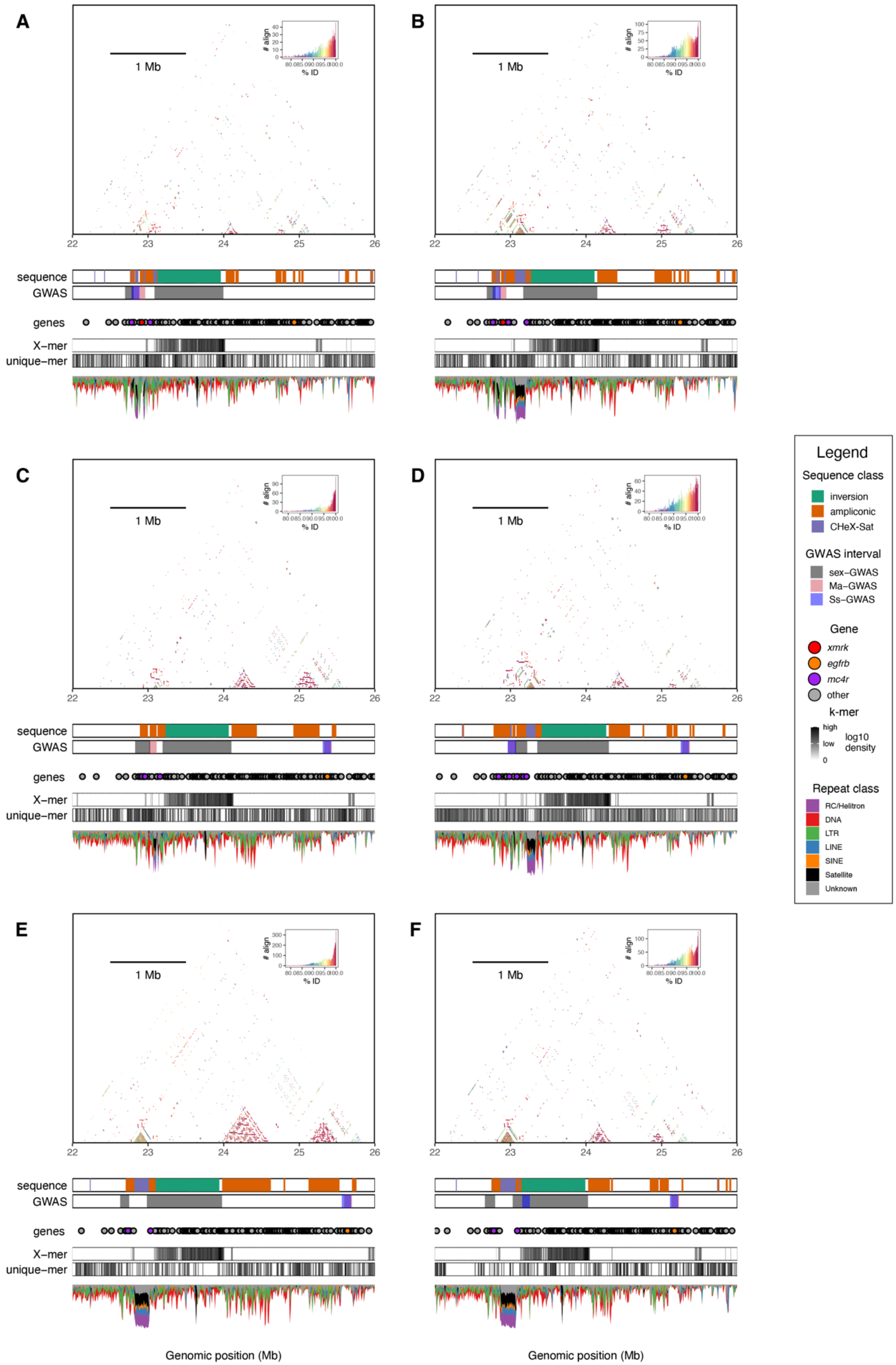
A
B
C
D
E
F
1 Mb
align
% ID
22
23
24
25
26
sequence
GWAS
genes
X–mer
unique–mer
Genomic position (Mb)
Legend
Sequence class
inversion
ampliconic
CHeX-Sat
GWAS interval
sex–GWAS
Ma–GWAS
Ss–GWAS
Gene
xmrk
egfrb
mc4r
other
k-mer
high
low
0
log10 density
Repeat class
RC/Helitron
DNA
LTR
LINE
SINE
Satellite
Unknown

Figure S10. Combined structural variant plots of all X chromosomes. (A) SspF01-h1 (*xmrk*), **(B)** Ss-M01-h1 (*xmrk*), **(C)** Ss-F01-h2 (non-*xmrk*), **(D)** Ss-M02-h2 (non-*xmrk*), **(E)** Ma-M01-h2 (non-*xmrk*), **(F)** WT-M01-h2 (non-*xmrk*). For the X chromosomes in this species, repeat content (ampliconic genes and satellites) and *xmrk* presence do not appear to be clearly linked. Several X chromosomes have non-mc4r ampliconic gene expansion on the other side of the inversion, with Ma-M01-h2 having the largest. Size and number of satellite blocks also vary among X chromosomes, with Ss-F01-h1 and Ss-F01-h2 having small satellite blocks, Ss-M01-h1 and Ss-M02-h2 having a more interspersed structure, and Ma-M01-h2 and WT-M01-h2 both having one very large block.

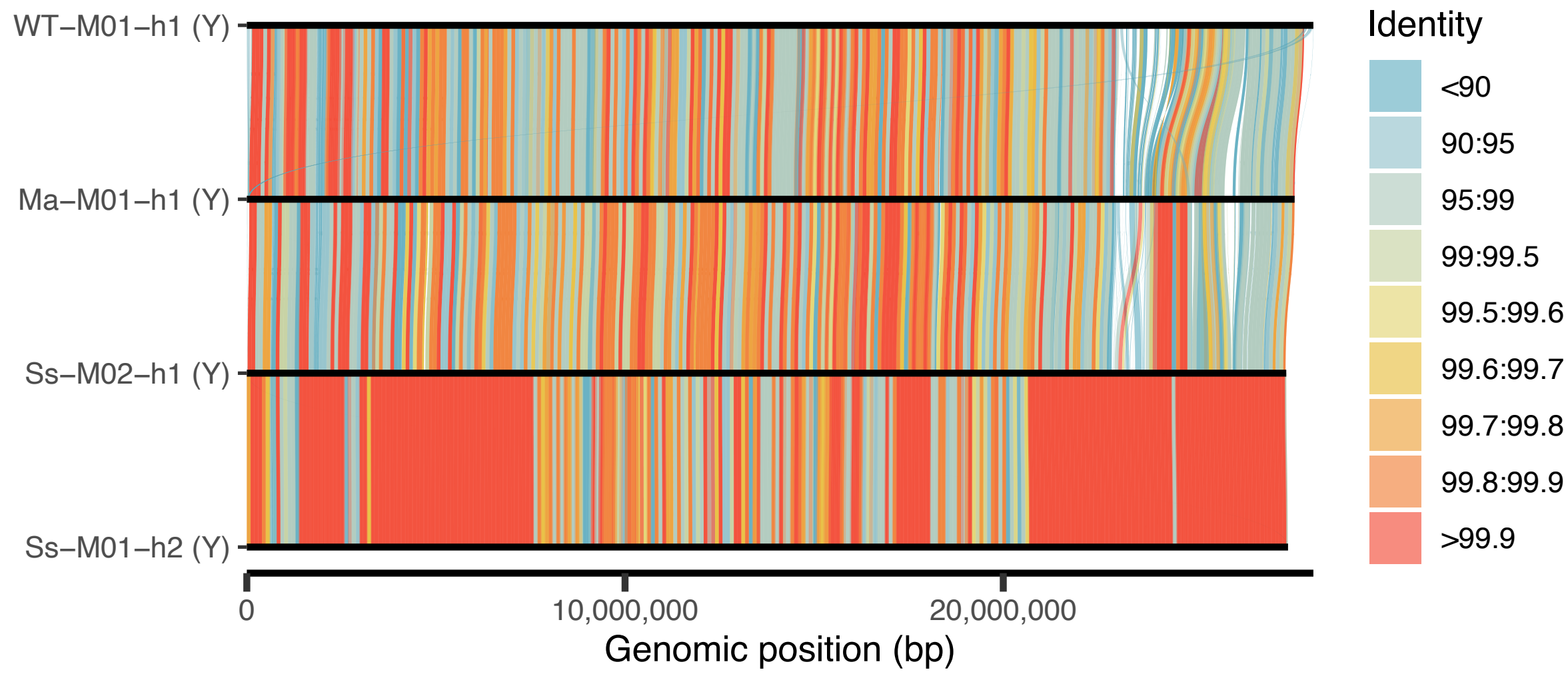

Figure S11. **The spotted side Y chromosomes are likely derived from close relatives.** SVbyEye alignments of *X. nezahualcoyotl* Y chromosomes colored by percent identity in 100kb bins. Note that these percent identity calculations also treat gaps in the alignment as non-matching basepairs. Ss-M02-h1 (Y) and Ss-M01-h2 (Y) were sourced from lab born males in the same tank. They have several megabase-long stretches of 100% identity, including at the end of the sex chromosome covering the sex determining region. This stretch of perfect alignment is interrupted only by a scaffold gap present in the Ss-M01-h2 assembly. These long stretches of perfect identity suggest that these two males are close (potentially first-degree) relatives, that share the sex-determining portion of the Y chromosome due to identity by descent, and therefore these two Y chromosomes should not be considered independent.

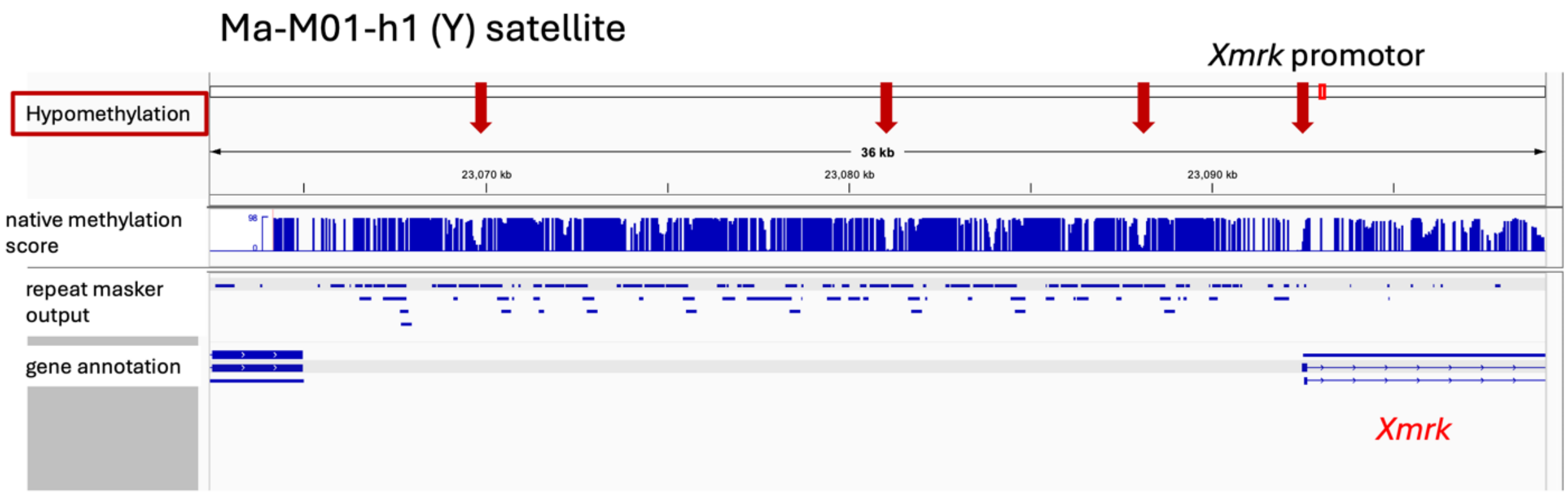

Figure S12. Methylation status within CHeX-sat sequences adjacent to *xmrk*. PB-CpG-tools methylation scores derived from native long-read data from adult male brain tissue of the marmoratus male. The composite satellite region is generally hypermethylated except for at the boundary of sub-elements annotated as Sat-chimeric and RC/Helitron4-chimeric. The *xmrk* promotor is also hypomethylated.

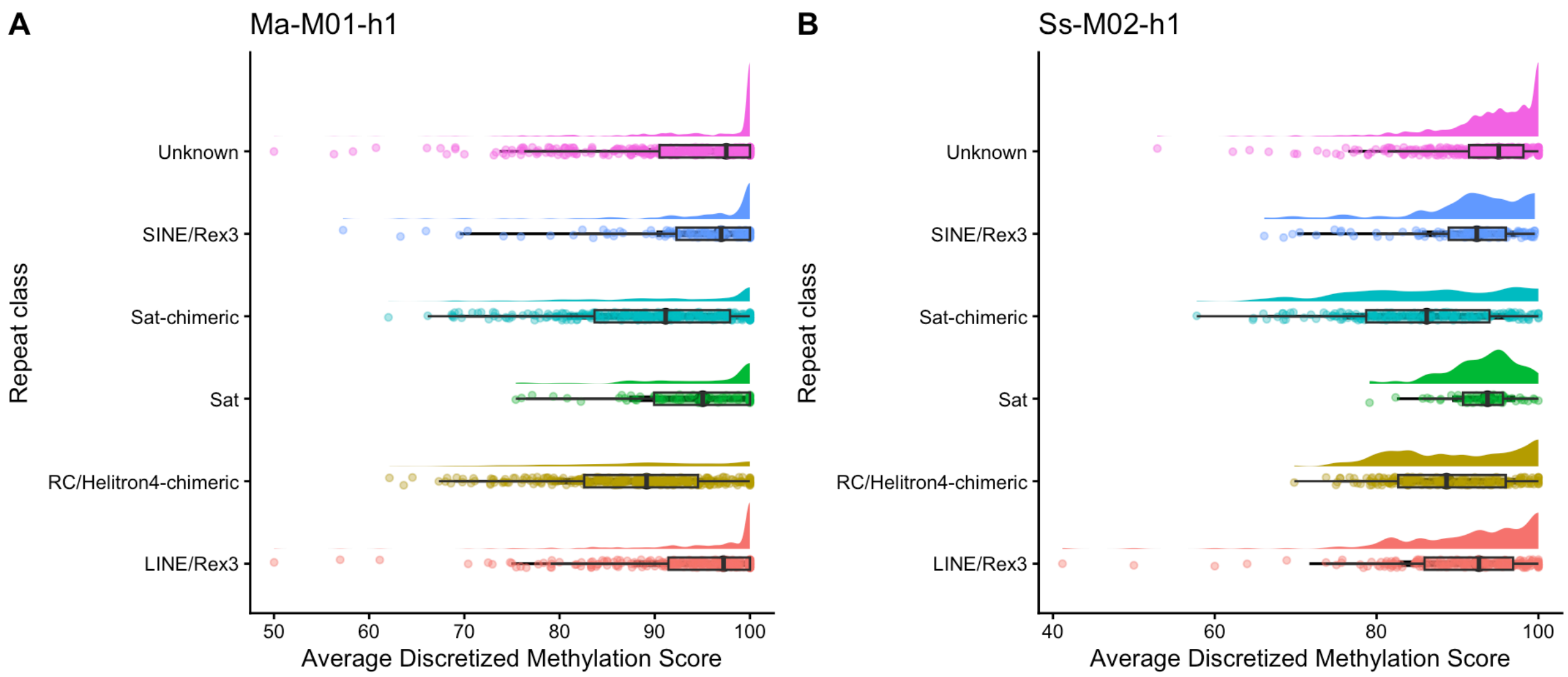

Figure S13. Methylation status within CHeX-sat sub-elements on the sex chromosome. PB-CpG-tools methylation scores derived from the CHeX-sat sequences from the Y chromosome of one marmoratus and one spotted side individual. Elements of the composite satellite show reduced methylation in sub-elements annotated as Sat-chimeric and RC/Helitron4-chimeric.

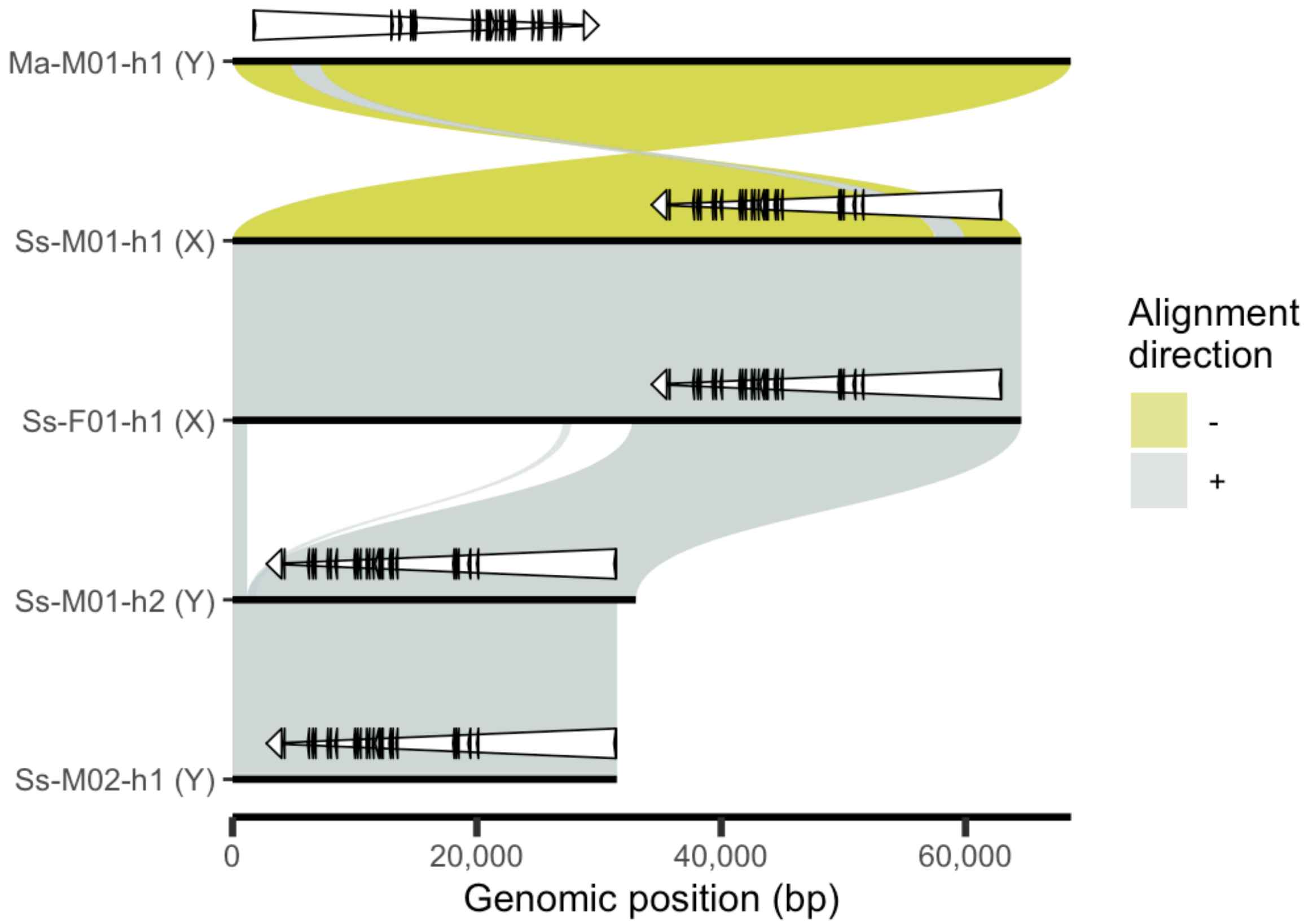

Figure S14. **SVbyEye alignments *X. nezahualcoyotl xmrk* sequencies colored by orientation.** The marmoratus haplotype is inverted relative to all other haplotypes and contains a nested 3 kb inversion within the first intron of *xmrk*. *xmrk* exons and the full transcript are both represented by white triangles.

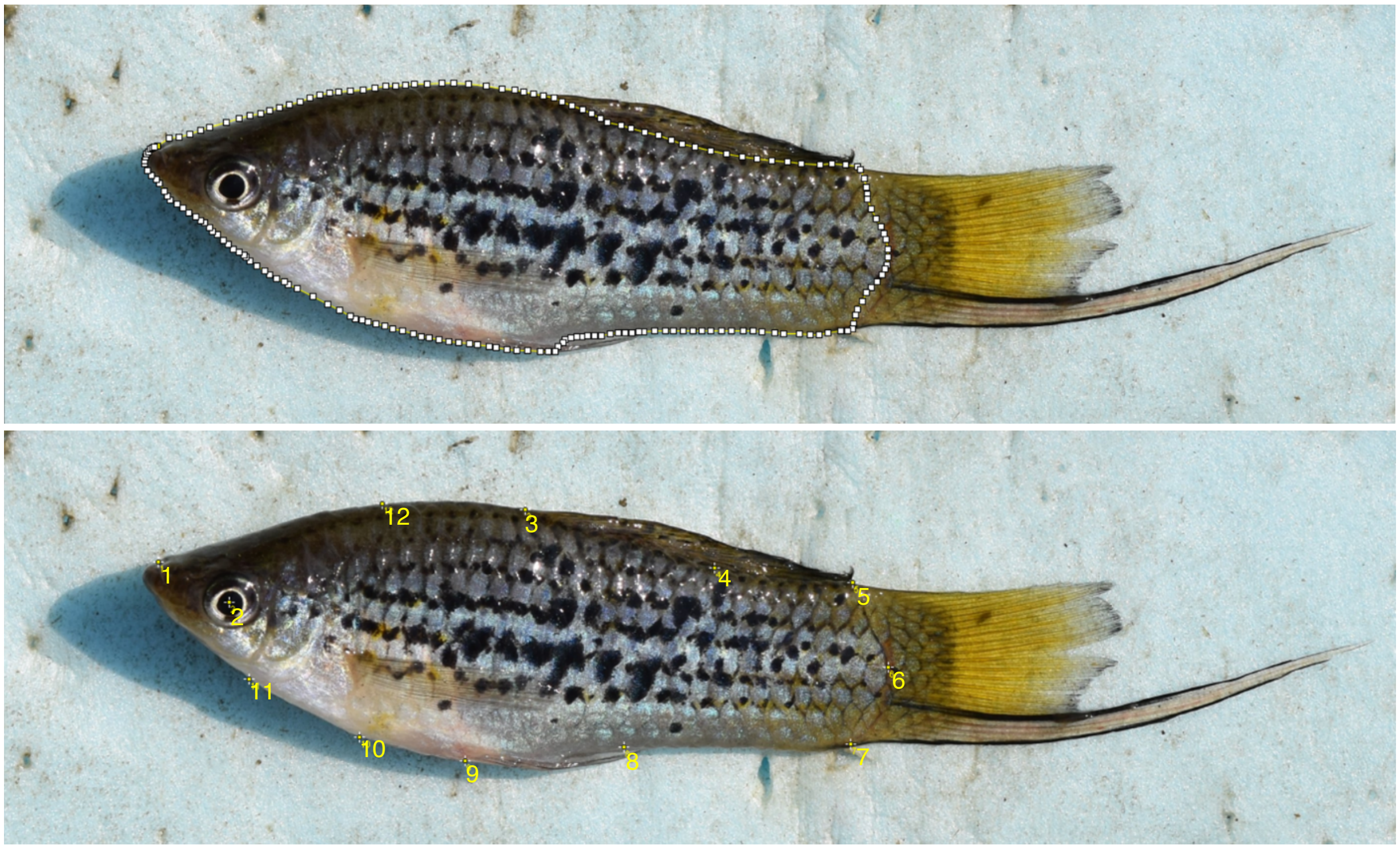

Figure S15. Reference images for recolorize/patternize workflow. One male was selected as a “reference” to align all images. Top shows the reference outline made using the polygon tool in FIJI. Bottom shows the 12 landmarks placed on every individual for alignment.

References

1. Rhie A, Nurk S, Cechova M, Hoyt SJ, Taylor DJ, Altemose N, et al. The complete sequence of a human Y chromosome. Nature. 2023 Sep;621(7978):344–54. doi:10.1038/s41586-023-06457-y
2. Brandt DYC, Aguiar VRC, Bitarello BD, Nunes K, Goudet J, Meyer D. Mapping Bias Overestimates Reference Allele Frequencies at the HLA Genes in the 1000 Genomes Project Phase I Data. G3 Genes|Genomes|Genetics. 2015 May 1;5(5):931–41. doi:10.1534/g3.114.015784
3. Ballouz S, Dobin A, Gillis JA. Is it time to change the reference genome? Genome Biol. 2019 Aug 9;20(1):159. doi:10.1186/s13059-019-1774-4
4. De Coster W, Weissensteiner MH, Sedlazeck FJ. Towards population-scale long-read sequencing. Nat Rev Genet. 2021 Sep;22(9):572–87. doi:10.1038/s41576-021-00367-3
5. Quan C, Lu H, Lu Y, Zhou G. Population-scale genotyping of structural variation in the era of long-read sequencing. Computational and Structural Biotechnology Journal. 2022 Jan 1;20:2639–47. doi:10.1016/j.csbj.2022.05.047
6. Aqil A, Gokcumen O. Read mismapping from segmental duplications drives spurious trans associations [Internet]. bioRxiv; 2025 [cited 2026 May 17]. p. 2025.10.08.681258. Available from: https://www.biorxiv.org/content/10.1101/2025.10.08.681258v1 doi:10.1101/2025.10.08.681258
7. Lovell JT, Rhein H, Sreedasyam A, Bentley N, Klein PE, Chatwin W, et al. Dissecting the regulatory and genomic drivers of the dichogamy determining G-locus in pecan [Internet]. bioRxiv; 2026 [cited 2026 May 19]. p. 2026.05.14.725045. Available from: https://www.biorxiv.org/content/10.64898/2026.05.14.725045v1 doi:10.64898/2026.05.14.725045
